# Plant polyphenols as inhibitors of NF-κB induced cytokine production—a potential anti-inflammatory treatment for Alzheimer's disease?

**DOI:** 10.3389/fnmol.2015.00024

**Published:** 2015-06-16

**Authors:** Niloo Karunaweera, Ritesh Raju, Erika Gyengesi, Gerald Münch

**Affiliations:** ^1^School of Medicine, University of Western SydneyPenrith, NSW, Australia; ^2^National Institute of Complementary Medicine, University of Western SydneyPenrith, NSW, Australia; ^3^Molecular Medicine Research Group, University of Western SydneyPenrith, NSW, Australia

**Keywords:** Alzheimer's disease, cytokine, neutraceuticals, inflammation, disease-modifying drug, treatment

## Chronic neuroinflammation and Alzheimer's disease

Alzheimer's disease (AD) is a neurodegenerative disorder that impacts the daily lives of many sufferers through memory loss as well as behavioral and cognitive changes. AD is the most common form of dementia. One in ten people over the age of 65, and around half of those over 85 have AD. AD can be divided into familial (early-onset) and sporadic (late-onset) cases, with the familial form (<1%) linked to mutations in three major genes (amyloid precursor protein, presenilin-1 and 2), and the sporadic form (>99% of cases) caused by a variety of genetic (e.g., apolipoprotein E), metabolic and environmental factors.

The AD brain is characterized macroscopically by cortical atrophy, caused by degeneration of the cholinergic axonal arborisation and shrinkage of the dendritic tree. Microscopically, amyloid beta peptide deposits (senile plaques) and neurofibrillary tangles are present in affected areas (Gil-Bea et al., 2012). AD is also characterized by chronic neuroinflammation, driven by activation of astroglia and microglia (Rosenblum, 2014). In addition, levels of pro-inflammatory mediators or cytokines which include chemokines, interferons, interleukins, lymphokines, and tumor necrosis factors are elevated in the brains of patients with AD (Latta et al., 2014). Furthermore, nuclear translocation of NF-κB and STAT-1α, transcription factors involved in pro-inflammatory gene expression, indicates the presence of a sustained pro-inflammatory process (Lawrence, 2009).

## Drug discovery difficulties faced in Alzheimer's disease

Drug discovery for AD has been strongly focused on β-amyloid (initially plaques, then soluble oligomers), as genetic evidence from the familial cases supported by the hypothesis that β-amyloid must be driving the disease process. Based on the “amyloid cascade hypothesis,” anti-amyloid therapies were hoped to deliver a cure for AD (Robinson et al., 2004). Unfortunately, numerous clinical trials with active and passive amyloid vaccines as well as ɣ-secretase inhibitors have failed (reviewed in Castello et al., 2014). Currently, there are no disease-modifying drugs available for AD. Consequently, alternative therapeutic targets, such as neuroinflammation have been suggested for the prevention and treatment of AD (Shi et al., 2013; Latta et al., 2014). As the expression of many pro-inflammatory cytokines is driven by the transcription factor NF-κB (Hoffmann et al., 2006), we propose that brain-permeable inhibitors of NF-κB signaling have the potential to prevent or slow down the progression of AD.

## Role of nuclear factor-κB (NF-κB) in inflammation

**Figure 1:**
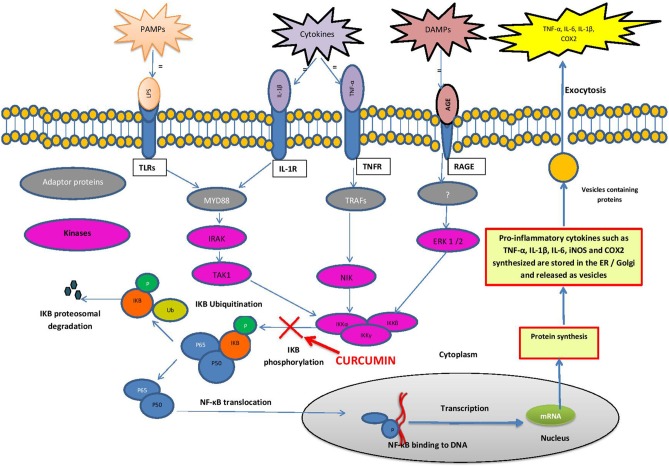
**Schematic diagram of the NF-κB pathway**. Cells express membrane receptors such as toll-like receptors (TLRs), interleukin-1β receptors (IL-1R), tumor necrosis factor receptors (TNFR) and receptor for advanced glycation end products (RAGE). These receptors recognize pro-inflammatory stimuli such as pathogen-associated molecular patterns (PAMPs), damage-associated molecular patterns (DAMPs) and cytokines. Ligand bound PAMPs, DAMPs and cytokines activate downstream adapter proteins such as myeloid differentiation primary response protein 88 (MyD88) and tumor necrosis factor associated factors (TRAF). MyD88 and TRAF activates specific protein kinases such as mitogen activated protein kinases (MAPK) such as IRAK, TAK1, NIK, and ERK 1/2. These kinases activate IκB kinases (IKKα, IKKβ, IKKγ) that phosphorylate IkB-α. In stimulated cells, phosphorylation of IkB leads to its dissociation from the complex, and its proteasomal degradation, allowing NF-kB to translocate to the nucleus, where it binds to specific DNA sequences present in the promoters of numerous target genes, encoding the pro inflammatory cytokines (e.g., IL-1, IL-2, IL-6, TNF-α), chemokines (e.g., IL-8, MIP-1α, MCP1, RANTES, eotaxin), adhesion molecules (e.g., ICAM, VCAM, E-selectin) as well as Cyclooxygenase-2 (Cox-2) and inducible nitric oxide synthase (iNOS).

Stimulation of microglia, the resident macrophages in the brain, initiates an inflammatory cascade, which involves NF-κB signaling. NF-κB is a ubiquitous transcription factor found in almost all animal cell types. NF-κB regulates the expression of many cytokines and chemokines, such as interferons, interleukins, lymphokines and tumor necrosis factors. Microglial cells express membrane receptors as toll-like receptors (TLRs), C-type lectin receptors (CLRs), nucleotide-binding oligomerization domain proteins (NLRs), the receptor for advanced glycation endproducts, RAGE and receptor for interferons and cytokines (Figuera-Losada et al., 2014). TLRs recognize pro-inflammatory ligands such as pathogen-associated molecular patterns (PAMPs) and damage-associated molecular patterns (DAMPs) (Figure 1). PAMPs include bacterial, fungal, parasitic, and viral molecules such as α- and β-glucans, viral RNA and DNA, flagellin, chitin, and microbial cell wall components (Figuera-Losada et al., 2014). DAMPs include blood-clotting factors, RNA, DNA and a variety of intracellular proteins released from damaged and dying cells (Figuera-Losada et al., 2014).

In unstimulated cells, NF-kB is present in the cytoplasm as an inactive heterodimer composed of two subunits, p50 and p65 (relA). The heterodimer forms a complex with the inhibitory proteins IkB-α or IkB-β, retaining it in the cytoplasm. When stimulated by a PAMP, DAMP or cytokine, it triggers a cascade of signaling events initiated by stimulation of adapter proteins in the cytoplasm. In the case of many TLRs, myeloid differentiation primary response protein 88 (MYD88) is such an adaptor protein (Mogensen, 2009; Figuera-Losada et al., 2014). MYD88 activates specific protein kinases downstream, such as mitogen activated protein kinase (MAPK) and IκB kinase which phosphorylate IkB-α. Phosphorylation of IκB-α and its proteasomal degradation leads to its dissociation from the NF-κB complex. NF-κB then translocates into the nucleus, where it binds to the promotor regions of specific genes (Brasier, 2006; Gilmore, 2006; Perkins, 2007). NF-κB then recruits other proteins (coactivators and RNA polymerase), which finally lead to the expression of many chemokines (e.g., IL-8, MIP-1α, MCP1, RANTES and eotaxin), proinflammatory cytokines (e.g., IL-1, IL-2, IL-6 and TNF-α), adhesion molecules (e.g., ICAM, VCAM, E-selectin) as well as inducible pro-inflammatory enzymes (COX-2 and iNOS), which exacerbate and perpetuate the inflammatory process (Barnes, 1997; Ghosh and Karin, 2002; Figuera-Losada et al., 2014).

## Plant polyphenols and their ability to prevent age-related degenerative diseases

Many dietary plants including fruits, vegetables and whole grains contain substantial amounts of polyphenols (Carlsen et al., 2010; Gunawardena et al., 2014). Polyphenols are characterized as compounds with phenolic structural features (Liu, 2004; Tsao, 2010). Dietary polyphenols have been suggested to aid in the prevention of degenerative diseases, including cancer, cardiovascular disease and neurodegenerative diseases such as AD due to their anti-inflammatory and anti-oxidant properties (Liu, 2004; Tsao, 2010). The high diversification of plant polyphenols have led to different ways of categorizing these naturally occurring compounds (Liu, 2004; Tsao, 2010). Polyphenols differ by their source of origin, chemical structure and biological effects (Liu, 2004; Tsao, 2010; Bellik et al., 2012a; Ebrahimi and Schluesener, 2012). They are secondary plant metabolites produced to aid in the defense mechanism against herbivores, insects, ultraviolet radiation, and microorganisms (Yoon and Baek, 2005; Tsao, 2010). More than 8000 plant polyphenols are currently known and among them more than 4000 flavonoids have been identified (Tsao, 2010). Some polyphenols lead to growth inhibition in laboratory animals (Ebrahimi and Schluesener, 2012). However, epidemiological data suggest that intake of small amounts of polyphenols in foods and beverages has a potent effect on reducing chronic diseases (Gotsis et al., 2015). There are studies that have investigated the effectiveness of fruit and vegetable rich diets such as the Mediterranean diet against degenerative diseases, indicating that these diets lead to a reduced incidence of degenerative and age-related diseases including cancer, cardiovascular diseases and neurodegenerative diseases (Calabrese et al., 2003; Yoon and Baek, 2005; Pan et al., 2010; Sofi et al., 2010; Bellik et al., 2012b; Vetrani et al., 2013; Gunawardena et al., 2014).

## Anti-inflammatory activity of plant polyphenols

Many plant polyphenolic compounds including curcumin, apigenin, quercetin, (*E*)-cinnamaldehyde and (*E*) -resveratrol have been shown to have anti-inflammatory activities in cell culture studies (Gautam and Jachak, 2009). Molecular targets of plant polyphenols acting as anti-inflammatory compounds include arachidonic acid (AA) dependent pathways and AA independent pathways. In the AA-dependent pathway, the anti-inflammatory effect of plant polyphenols is related to their ability to inhibit COX (the isoform Cox-2, also regulated by NF-κB), which converts AA into prostaglandins. AA-independent pathways involve AA signaling through nuclear factor-kappa B (NF-κB) (Yoon and Baek, 2005).

Polyphenols have been shown to interfere at two specific sites in the pathway leading from receptors to NF-kB. Some polyphenols inhibit kinases by inhibiting their phosphorylation or ubiquitination and therefore prevent the subsequent degradation of IkB (Ruiz and Haller, 2006). This prevents NF-κB translocation into the nucleus and transcription of pro-inflammatory cytokines. Additionally, inhibition of the interaction of NF-κB subunits with target DNA has also been proposed as a mode of action of anti-inflammatory polyphenols (Ruiz and Haller, 2006). Both modes of action ultimately lead to the inhibition of expression of various NF-κB regulated pro-inflammatory proteins (cytokines, chemokines) and enzymes (iNOS, COX-2).

Among the polyphenols that have been shown to modulate pro-inflammatory gene expression are curcumin (Jobin et al., 1999), apigenin (Wang et al., 2014), resveratrol (Kundu et al., 2006), quercetin (Endale et al., 2013), silymarin (Saliou et al., 1998) cinnamaldehyde (Reddy et al., 2004), pathenolode (Saadane et al., 2007), ergolide (Chun et al., 2007), 2β,5-epoxy-5,10-dihydroxy-6α-angeloyloxy-9β-isobutyloxy-germacran-8α,12-olide (Lee et al., 2011), andalusol (Heras et al., 1999), ent-kaur-16-ene-19-oic acid (Wu et al., 2013), kamebanin (Hwang et al., 2001), kamebacetal A (Hwang et al., 2001), kamebakaurin (Hwang et al., 2001), excisanin A (Hwang et al., 2001), hypoestoxide(Ojo-Amaize et al., 2001), helenalin (Lyss et al., 1997), pristimerin (Tiedemann et al., 2009), epigallocatechin gallate (Kim et al., 2010), avicin (Haridas et al., 2001), capsaicin (Singh et al., 1996), and oleandrin (Sreenivasan et al., 2003), just to name a few. In this opinion paper, we will focus on curcumin as the prime example as the manuscript format does not allow to elaborate on the specific targets of each of the compounds in the NF-κB pathway.

## Curcumin is a prototype inhibitor of NF-κB signaling acting upstream of IκB

Curcumin occurs naturally in the root of plant *Curcuma longa* which belongs to the Zingiberaceae family. The root of this plant is powdered and used in cooking for centuries in Asian countries. It has also been used extensively in traditional Indian and Chinese medicine to treat diabetic wounds, hepatic disorders, sinusitis and rheumatism (Chainani-Wu, 2003).

Curcumin has been shown to have various anti-inflammatory properties. Curcumin has a broad cytokine-suppressive anti-inflammatory action, it down-regulates the expression of cyclooxygenase-2 (COX-2), lipoxygenase, and inducible nitric oxide synthase (iNOS) enzymes. Curcumin also inhibits the production of the pro-inflammatory cytokines such as TNF-α, IL-1, -2, -6, -8, and -12, and the neurotoxic factors in LPS-stimulated monocytes and alveolar macrophages (Abe et al., 1999). Furthermore, the pharmacokinetic properties of curcumin are also favorable. Curcumin crosses the brain-blood-barrier and bioavailable curcumin preparations (orally applied) can achieve therapeutic concentrations in the brain. For example, brain levels of the curcuminoids reached concentrations of up to 3 μM for curcumin, and up to 6 μM for tetrahydrocurcumin (TC). The EC_50_ value for iNOS mRNA inhibition *in vivo* was 1.2 and 0.7 μM for curcumin and TC, respectively.

Inhibition of NF-κB signaling is the best described mechanism of anti-inflammatory action of curcumin (Ambegaokar et al., 2003; Shakibaei et al., 2007). It has been shown that curcumin inhibits the phosphorylation and degradation of IκBα and the subsequent translocation of the p65 subunit of NF-κB to the nucleus (Singh and Aggarwal, 1995). Although the exact target is not known, the study indicates that curcumin interferes with the NF-κB pathway upstream of IκBα phosphorylation (Figure 1). In a further study in intestinal epithelial cells, curcumin has also been shown to block a signal leading to IKK activation (Jobin et al., 1999).

## Conclusions

We suggest that inhibitors of NF-kB activation should have the potential to prevent and delay the onset or even treat Alzheimer's disease (Bremner and Heinrich, 2002). However, clinical trials are needed to confirm the effectiveness of some of these plant-based inhibitors of NF-κB pathway as a treatment option for Alzheimer's disease. Nonetheless, in preparation of those trials, critical pharmacokinetic parameters have to be addressed beforehand. Comprehensive investigation models with multidisciplinary approach, including epidemiological, clinical and cellular molecular studies should be employed to ensure that the doses extrapolated from the *in vitro* data determined in cell cultures have pharmacological relevance (e.g., the tissue concentration matching the EC_50_ values). Their efficacy has to be tested in animal models of AD, which exhibits some degree of chronic inflammation generated by the pathological deposits such as beta-amyloid or tau, or in a “pure” animal model of chronic neuroinflammation such as the IL-1β or IL-6 overexpressing mouse (Ebrahimi and Schluesener, 2012; Millington et al., 2014). If these animal studies are successful, and show not only a decrease in NF-κB regulated pro-inflammatory mediators, but also an improvement in cognition and memory, then plant polyphenols might have the potential to be the first disease modifying drugs for the treatment of Alzheimer's disease.

### Conflict of interest statement

The authors declare that the research was conducted in the absence of any commercial or financial relationships that could be construed as a potential conflict of interest.
